# Early Consequences of Pectus Excavatum Surgery on Self-Esteem and General Quality of Life

**DOI:** 10.1007/s00268-018-4526-9

**Published:** 2018-02-06

**Authors:** W. P. Zuidema, J. W. A. Oosterhuis, G. W. Zijp, S. M. van der Heide, A. F. W. van der Steeg, L. W. E. van Heurn

**Affiliations:** 1 Pediatric Surgical Center Amsterdam, Emma Children’s Hospital AMC, VU-University Medical Center, De Boelelaan 1117, 1081 HV Amsterdam, The Netherlands; 2 Department of Surgery, Haaglanden Medical Center, The Hague, The Netherlands; 3 Pediatric Surgery, Juliana Children’s Hospital/Haga-Hospital, The Hague, The Netherlands; 4 Cardio-Thoracic Surgery, Radboud University Medical Center, Nijmegen, The Netherlands; 5 Center of Research on Psychology in Somatic Disease (CoRPS), Tilburg University, Tilburg, The Netherlands

## Abstract

**Background:**

An early observation after chest wall correction is direct inspection from the PE patient of their “new” thorax. Changes in self-perception may give raise to other psychological adaptations. The aim of this study was to evaluate the early changes in the fields of self-esteem, body image and QoL.

**Methods:**

Prospective observational longitudinal multicenter cohort study. Self-esteem, emotional limitations and general health were assessed using the Child Health Questionnaire (CHQ) in patients under 18 and the World Health Organization Quality of Life Questionnaire-bref (WHOQOL-bref) was used for body image, psychological domain and overall QoL in patients over 16 years of age. Measurements were taken before surgery (T1) and 6 weeks (T2), and 6 months thereafter (T3).

**Results:**

Scores on post-operative self-esteem were significantly higher compared with scores pre-operatively (*p* < 0.007). Also body image, psychological domain and emotional limitations showed significant improvement, respectively *p* < 0.001, *p* < 0.001, and *p* < 0.016. Significant improvement in the first three components was mainly achieved in the first 6 weeks post-operative. In emotional limitation, however, the largest change was between 6 weeks and 6 months. Overall quality of life in the WHOQOL-bref and general health domain in the CHQ showed no significant improvement in relation to the pre-operative scores.

**Conclusion:**

Post-operative PE patients after Nuss procedure showed an improved body image, increased self-esteem and increased psychological resilience in the first 6 months, with the most marked change in the first 6 weeks. Also emotional limitations changed significantly over time. The changes were not large enough to influence general QoL or general health significantly.

## Introduction

Pectus surgeons all notice that the first thing patients do after surgical correction of their pectus excavatum (PE) is looking at their chest wall, to see how it is changed. Thus, their appearance is important and is expected to have effect on their self-esteem or body image.

Pectus excavatum is the most important anterior chest wall deformity and affects predominantly males. The estimated incidence of PE is approximately 1 in 400 youngsters [1]. The primary complaint is cosmesis although a substantial part of the patients also complains of physical impairments, especially shortness of breath during exercise.

There are in general two surgical procedures used for correction, the open (Ravitch)—and the minimally invasive (MIPRE or Nuss) procedure. Both procedures have been reported to give good cosmetic results [2]. Studies reporting additional physical improvement after correction of PE are becoming more frequent [3–9].

The cosmetic and physical issues may give rise to a decreased self-esteem and quality of life (QoL), especially in adolescents who are vulnerable to group pressure [10]. These are important reasons for the surgeon to take into account in deciding about a possible operative procedure. The different variables are weighed specific in every patient. These considerations can give rise to situations were complaints about cosmesis even with an objectively mild pectus can lead to surgical correction [11].

Quality of life is defined by the World Health Organization as ‘an individual’s perception of his/her position in life in context of the culture and value systems in which he/she lives and in relation to his/her goals, expectations, standards and concerns’ [12]. This is a definition covering wide areas of personal functioning. The QoL is different for every patient since it is a subjective feeling and in the end is all about satisfaction with one’s physical capabilities and appearance.

The present study aims to evaluate the early changes in cosmesis and its possible effects especially on body image after surgical PE correction. Since body image is a part of one’s self-esteem, the latter may also be influenced. Even an effect on general QoL is possible although it is questionable if this effect would be large enough to be measurable, since QoL relates to so much more than body image and self-esteem.

We hypothesized that pectus excavatum surgical correction would have an early positive effect on body image and self-esteem, but no significant measurable effect on general QoL.

## Materials and methods

### Patients

All consecutive patients who were referred with a PE to the outpatient clinic of one of the five participating centers (AMC, VUMC, UMCG, Juliana Children’s Hospital/Haga-Hospital, Radboud UMC) were asked to participate in this study. Patients younger than 12 years of age were not eligible for correction at our institutions and therefore did not participate. Excluded were also patients or parents with insufficient knowledge of the Dutch language. Those with associated connective tissue diseases were allowed to participate in the study.

Patients under the age of sixteen gave informed consent as did their parents. All patients over the age of sixteen gave informed consent. The medical ethics committee approved the study.

In a previous paper, we investigated the first small group of this study population on the association between pain and QoL [13].

### Surgery

In all study, patients with PE the Nuss procedure was performed [14]. Surgery was performed by dedicated pediatric or thoracic surgeons. The operative technical procedure was similar in all centers. Post-operative pain management was done with patient controlled epidural analgesia or patient controlled intravenous analgesia using morphine and occasionally ketamine. When possible this was changed after 3 days to oral pain medication.

### Questionnaires

Patients were divided into three groups based upon age, being younger than 16 years, 16–18 years and older than 18 years of age. Questionnaires used differed per age group; this was necessary to meet the validation criteria for the different questionnaires which are limited to age group.

Pre-operative questionnaire was commenced on the last outpatient clinic visit prior to surgery. Post-operative written questionnaires were sent to the patients’ home address on the calculated date. If no direct response was received, a reminder by either mail or telephone was used. Measurement moments were pre-operatively, 6 weeks and 6 months post-operative.

The body image and psychological domain were determined using the World Health Organization Quality Of Life Questionnaire-bref. This is the short version of the WHOQOL-100 [15]. It consists of questions assessing QoL in four domains being physical health, psychological health, social relationships, and environment and a general evaluative facet (overall quality of life and general health). For the purpose of our study, a complete facet of the WHOQOL-100 has been added to the WHOQOL-bref being the facet body image. Items are scored on a four-point Likert scale. Higher scores indicate a better QoL.

Self-esteem, emotional limitations, and general health were scored using the Child Health Questionnaire-87. It is a generic QoL assessment tool that has good reliability and validity [16]. This questionnaire covers the physical, emotional, and social well-being of children between the age of 8 and 18 years. Self-esteem as well as emotional limitations and general health are domains of the CHQ-87. In the domain, self-esteem one question is specific for body image perception of the patient. Items are scored using a four to six point Likert scale and converted to a 0–100 point continuum, with higher scores indicating a better QoL. Norm values of the Dutch population are available and allow for comparison with ‘healthy’ children [17].

Quality of life was assessed using the Dutch version of the CHQ-87 in patients younger than 16 and between 16 and 18 years of age and with the short version of the World Health Organization Quality of Life assessment instrument (WHOQOL-bref) in patients between 16 and 18 years and older than 18 years of age. This implies that some patients completed both CHQ and WHOQOL-bref at all three measurement moments. In these cases, both questionnaires were included in the analyses to establish whether they would come to the same results.

### Statistical analysis

Data analyses were conducted using IBM SPSS 23 software (SPSS Inc. Chicago, IL, USA). Descriptive statistics for variables of interest in this study are presented as percentage, means and SDs. Comparison between scores at measurement moment T1 and T2, T1 and T3, and T2 and T3 for the enlisted variables from the study group were calculated using the paired Student’s *T* test. The General Linear Model (GLM) was used for comparison of all three measurements. The cutoff point for significance was set at *p* < 0.05.

## Results

Between October 2011 and December 2016 in an ongoing study, 131 patients were included who had measurements on all three moments (pre-operative, 6 weeks and 6 months post-operative). To be able to perform the analyses, only patients were included that completed the same questionnaire at all three moments.

They consisted of 113 males and 18 females of whom 82 patients had only CHQ scores recorded at these three points in time, 23 patients only the WHOQOL-bref and 26 patients completed both the WHOQOL and the CHQ at all three measurement moments. All patients underwent a Nuss procedure because of a PE. The mean age was 16.1 years (SD 2.3) with 17 patients being older than 18 years of age and the youngest patient being 12 at time of surgery. For general patient characteristics, see Table 1.

**Table 1:** Patient characteristics

Age (years)	16.1 (2.3)^a^
Hospital admission (days)	6.7 (1.8) days
Operation time (min)	55.5 (23.1) min
Minor complications <6 weeks	0.7 (0.4)^a^
Major complications <6 weeks	0.1 (0.3)^a^
BMI	18.7 (2.3)^a^

^a^Scores are represented in means (SD)

Scores on the WHOQOL-bref showed an overall significant improvement for the facet body image between pre-operative value 12.1 (SD 3.6) and 6 weeks past surgery 15.7 (SD 2.6) and at 6 months 16.1 (SD 2.9) (*p* < 0.001); (see Table 2). Further analyses showed a significant improvement between pre-operative and 6 weeks post-operative (*p* < 0.001) but not between 6 weeks and 6 months post-operative (*p* = 0.106).

**Table 2:** Comparison between repeated scores on WHOQOL and CHQ for T1, T2 and T3

Measurement moment	T1	T2	T3	*p* value (overall)
WHOQOL				
Facet body image	12.1 (3.6)	15.7 (2.6)	16.1 (2.9)	<0.001
Psychological domain	13.9 (2.4)	15.0 (2.6)	15.4 (2.6)	<0.001
Overall QoL	8.1 (1.2)	8.3 (1.5)	8.3 (1.3)	0.503
CHQ				
Self-esteem	72.5 (13.4)	75.8 (13.6)	75.4 (14.3)	0.007
Emotional limitation	92.9 (13.9)	90.0 (18.0)	94.3 (13.4)	0.016
General health	77.7 (18.5)	75.0 (18.0)	76.2 (19.6)	0.325

T1 stands for pre-operative. T2 for 6 weeks. T3 for 6 months. Scores are represented in means (SD). Concerning scores: a higher score represents improvement. The General Linear Model (GLM) was used for (overall) comparison of all three measurements. The cutoff point for significance was set at *p* < 0.05

The psychological domain of the WHOQOL-bref also showed an overall significant improvement at 6 weeks and 6 months compared to pre-operative measurements (*p* < 0.001), of which the significant improvement fell in the first time interval (*p* = 0.002) and not in the second time period between 6 weeks and 6 months (*p* = 0.087).

Scores on post-operative self-esteem assessed with the CHQ were overall significantly higher compared to scores pre-operatively (*p* = 0.007). Comparison between the two time intervals after surgery showed the increase in self-esteem in the first 6 weeks (*p* = 0.002) and not between 6 weeks and 6 months (*p* = 0.752).

The emotional limitations domain also showed a significant improvement (*p* = 0.016), in which the second time interval was more important than the first (*p* = 0.060 vs. *p* = 0.009). Overall quality of life in the WHOQOL-bref and general health domain in the CHQ showed no significant improvement in relation to the pre-operative scores.

## Discussion

The primary goal of pectus correction is improvement of self-esteem, body image, quality of life and sometimes physical impairment.

Other studies have shown a long-term positive relationship between surgical correction of a thoracic wall deformity and improvement of body image [3]. Concerning QoL, however, a small study by. Lam et al. [2] with only 11 Nuss patients included showed no return to levels of QoL comparable to their peers in the long term. On the other hand, in the group of Kim et al. [18] consisting of 39 Nuss patients, there was improvement of QoL in the long run. Our assumption that on the short term (i.e., 6 weeks and 6 months after surgery), the more focused scores as body image, self-esteem, and psychological functioning would show fast improvement was confirmed. The largest changes in body image, self-esteem and psychological domain take place in the direct post-operative period of the first 6 weeks, whereas the major changes in emotional limitation take place between 6 weeks and 6 months. Probably, the lower score on 6 weeks (general health and emotional limitations) is due to the negative effect of pain and physical recovery time in the first weeks post-operatively. However, the influence on body image, self-esteem and psychological functioning was not so large that it gave a significant improvement within the wide definition of general QoL. Measurements with a longer follow-up are necessary to find out whether QoL does improve on the long run for our patients.

Unfortunately, there are no studies who have measured individuals with PE who were not operated with regard to evolution in body image, self-esteem, psychological profile and QoL over time. Comparison of effects in the PE patients can only be related to references score of healthy adolescents.

There are whoever studies of patients with cosmetically visible disorders who can be used for reference. Patients treated surgically for gynecomastia showed a long-term improvement in self-esteem and satisfaction [19]. Another study concerned children with prominent ears who underwent otoplasty. Follow-up measurements showed reduced psychological problems post-operative and improved QoL [20] and improved self-esteem [21]. Young transplant patients showed variation in their developed self-esteem where long waiting lists, health status and female sex can negatively influence self-esteem [22, 23].

Since the Nuss operation is most often carried out in adolescents, it is fair to say they have a whole life ahead of them. The adolescent period is one in which important changes take place on biological and chemical changes of the body, but also emotional, sexual and social changes take place. This transition period in life between childhood and adulthood forms the basis of the latter one, and therefore, experiences in this period are of great influence in later life. One important area is self-esteem, and it is related to a personal evaluation of oneself that influences behavior, wherein a positive self-esteem leads to positive affect and more capability to cope with life and its challenges later on [22, 23].

In particular, because restoring self-esteem may have such an important positive effect in later life, the risk of surgery may be worth it.

## References

[1] Shamberger RC (1996). Congenital chest wall deformities. Curr Probl Surg.

[2] Lam MWC, Klassen AF, Montgomery CJ (2008). Quality-of-life outcomes after surgical correction of pectus excavatum: a comparison of the Ravitch and Nuss procedures. J Ped Surg.

[3] Kelly RE, Cash TF, Shamberger RC (2008). Surgical repair of pectus excavatum markedly improves body image and perceived ability for physical activity: multicenter study. Pediatrics.

[4] Krasopoulos G, Dusmet M, Ladas G (2006). Nuss procedure improves the quality of life in young male adults with pectus excavatum deformity. Eur J Cardiothorac Surg.

[5] Tang M, Nielsen HH, Lesbo M (2012). Improved cardiopulmonary exercise function after modified Nuss operation for pectus excavatum. Eur J Cardiothorac Surg.

[6] Maagaard M, Tang M, Ringgaard S (2013). Normalized cardiopulmonary exercise function in patients with pectus excavatum three years after operation. Ann Thorac Surg.

[7] Acosta J, Bradley A, Raja V (2014). Exercise improvement after pectus excavatum repair is not related to chest wall function. Eur J Cardiothorac Surg.

[8] Sigalet DL, Montgomery M, Harder J (2003). Cardiopulmonary effects of closed repair of pectus excavatum. J Pediatr Surg.

[9] Maagaard M, Heiberg J (2016). Improved cardiac function and exercise capacity following correction of pectus excavatum: review of current literature. Ann Cardiothorac Surg.

[10] Steinmann C, Krille S, Mueller A (2011). Pectus excavatum and pectus carinatum patients suffer from lower quality of life and impaired body image: a control group comparison of psychological characteristics prior to surgical correction. Eur J Cardiothorac Surg.

[11] Jaroszewski D, Notrica D, McMahon L (2010). Current management of pectus excavatum: a review and update of therapy and treatment recommendations. J Am Board Fam Med.

[12] WHOQOL group (1994). Development of the WHOQOL: rationale and current status. Int J Ment Health.

[13] Zuidema WP, van der Steeg AFW, Oosterhuis JWA (2014). The influence of pain: quality of life after pectus excavatum correction. Open J Pediatr.

[14] Nuss D, Kelly RE, Croitoru DP (1998). A 10-year review of a minimally invasive technique for the correction of pectus excavatum. J Pediatr Surg.

[15] WHOQOL group (1998). Development of the world health organization WHOQOL-Bref quality of life assessment. Psychol Med.

[16] Landgraf JM, Abetz L, Ware JA (1996). The CHQ user manual.

[17] Raat H, Landgraf JM, Bonsel GJ (2002). Reliability and validity of the child health questionnaire-child form (CHQ-CF87) in a Dutch adolescent population. Qual Life Res.

[18] Kim HK, Shim JH, Choi YH (2011). The quality of life after bar removal in patients after the nuss procedure for pectus excavatum. World J Surg.

[19] Fricke A, Lehner GM, Stark GB (2017). Long-term follow-up of recurrence and patient satisfaction after surgical treatment of gynecomastia. Aesthet Plast Surg.

[20] Songu M, Kutlu A (2014). Long-term psychosocial impact of otoplasty performed on children with prominent ears. J Laryngol Otol.

[21] Niemelä BJ, Hedlund A, Andersson G (2008). Prominent ears: the effect of reconstructive surgery on self-esteem and social interaction in children with a minor defect compared to children with a major orthopedic defect. Plast Reconstr Surg.

[22] Frieson TC, Frieson CW (1996). Relationship between hope and self-esteem in renal transplant recipients. J Transpl Coord.

[23] de Castro EK, Moreno-Jiménez B, Rodríguez-Carvajal R (2007). Psychological well-being in adults transplanted in childhood. Pediatr Transpl.

